# Training of executive functions in healthy elderly: Results of a pilot study

**DOI:** 10.1590/S1980-57642012DN06010006

**Published:** 2012

**Authors:** Thaís Bento Lima-Silva, Aline Teixeira Fabrício, Laís dos Santos Vinholi e Silva, Glaúcia Martins de Oliveira, Wesley Turci da Silva, Priscilla Tiemi Kissaki, Anna Pereira Fernandes da Silva, Tamiris Fessel Sasahara, Tiago Nascimento Ordonez, Thalita Bianchi de Oliveira, Flávia Ogava Aramaki, Adriana Buriti, Mônica Sanches Yassuda

**Affiliations:** 1 Bachelor's Degree in Gerontology, School of Arts, Sciences and Humanities, University of São Paulo (USP), São Paulo SP, Brazil; 2 Undergraduate Student in Gerontology, School of Arts, Sciences and Humanities, USP; 3 Psychologist, Santa Marcelina Hospital, São Paulo SP, Brazil; 4 Associate Professor in Gerontology, School of Arts, Sciences and Humanities, USP

**Keywords:** elderly, executive functions, cognitive training

## Abstract

Executive functions (EF) refer to the cognitive skills necessary to formulate a goal, plan, execute plans effectively, and to perform self-monitoring and self-correction. Several aspects of EF change during the normal aging process.

**Objectives:**

To train skills associated with executive functions in the elderly and to detect possible impact on objective EF tests and self-reports of functional status.

**Methods:**

A cross-sectional study involving an intervention and pre and post testing was carried out. Study participants included 26 seniors assigned to an experimental group (EG) and given six sessions of cognitive intervention, and 17 seniors assigned to a control group (CG) who completed pre and post testing only. All participants were enrolled in an Open University for the Third Age. The following tests were used to measure outcome: the Mini-Mental State Examination (MMSE), the Geriatric Depression Scale (GDS), the Story subtest of the Rivermead Behavioral Memory Test (RBMT) (versions A and B), semantic verbal fluency fruit category, and verbal fluency with phonological constraints (FAS), WAIS-III Digit Span, Clock Drawing Test (CDT), Trail Making Part A and the Pfeffer Functional Assessment Questionnaire (PFAQ). Delta scores were calculated (post-test score minus pretest score) to assess the impact of the intervention.

**Results:**

In the post test, the CG showed significant improvement on the RBMT Story recall and Digit Span but a decline in verbal fluency. The EG remained stable in terms of pre and post test scores.

**Conclusions:**

The intervention did not enhance performance on the EF tests. It is noteworthy that the EG received only a small number of sessions which may not have been sufficient to generate improvement. Alternatively, the lack of group differences observed could be associated to participation in other workshops offered at the university.

## Abstract (pt)

As funções executivas (FE) representam as capacidades cognitivas necessárias para formular um objetivo, planejar, executar planos de modo eficiente, monitorizar-se e autocorrigir-se. As FE sofrem alterações durante o processo de envelhecimento normal.

**Objetivos:**

Treinar habilidades relacionadas às funções executivas em idosos e detectar impactos em testes objetivos de FE e em autorrelato de desempenho funcional.

**Métodos:**

Trata-se de um estudo transversal, com intervenção e pré e pós testagem. Participaram do estudo 26 idosos que compuseram o grupo experimental (GE) que receberam intervenção cognitiva de seis sessões e 17 idosos do grupo controle (GC) que completaram apenas pré e pós testagem, matriculados em atividades em uma Universidade Aberta à Terceira Idade. Como medida de eficácia foram usados o Mini-Exame do Estado Mental (MEEM), Escala de Depressão Geriátrica (EDG), o subteste História do Teste Comportamental de Memória de Rivermead (versões A e B), fluência verbal categoria semântica frutas (FV), e com restrição fonológica (FAS), Dígitos da bateria WAIS-III, o Teste do Desenho do Relógio (TDR), Trilha A e o Questionário de Avaliação Funcional de Pfeffer (PFAQ). Para a avaliação do impacto da intervenção foram calculados deltas (escore do pós-teste menos o escore do pré-teste).

**Resultados:**

No pós-teste o GC apresentou melhora no resgate da História e no desempenho nos Dígitos, entretanto, apresentou pior desempenho em fluência verbal. A pontuação do GE permaneceu inalterada.

**Conclusões:**

Os dados sugerem que a intervenção não gerou impacto nos testes associados ao conceito de FE. Destaca-se que os idosos do GE receberam um número limitado de sessões que pode não ter sido suficiente para gerar alterações. Alternativamente, a ausência de diferença entre os grupos pode estar associada à participação em outras oficinas oferecidas na universidade.

## INTRODUCTION

Executive functions (EF) refer to abilities involved in formulating goals, planning, executing plans effectively, and self-monitoring and correction. The primary difference between EFs and other cognitive function is that the latter are related to "what" or "how much" a person knows.^1^ With EF however, the focus is more on "how" an individual goes about performing tasks. These functions encompass the skills that enable individuals to successfully become engaged in independent, objective and self-monitored behavior, and thus involve the more complex aspects of human cognition.^2^

More specifically, executive functions allow the ability to plan and develop strategies to achieve goals, a process calling for behavioral flexibility, an ability to integrate details coherently and manage multiple sources of information, in coordination with the use of previously acquired knowledge.^3^ The executive system is also responsible for adapting behavior in order to solve problems of everyday living.^4^

Executive functioning can be represented by four basic components: volition, planning, purposeful action and effective performance. Volition is a term referring to the ability to formulate goals and form intentions, motivation and self-awareness. Planning involves conceptual ability and abstraction, forward thinking, decision-making, the capacity to build steps and sequences, create alternatives, ponder, make choices and sustain attention. With regard to purposeful action, this denotes the translation of an intention into a useful, productive activity and requires the ability to initiate, maintain, switch and interrupt complex behavioral sequences in an integrated and ordered manner, and also includes flexibility.^1^

Furthermore, the performance of several other cognitive functions relies on working memory (WM) and in the context of cognitive training (CT) these functions depend on stimulation of WM in order to improve.^5^ Therefore, decline in this function can compromise performance on everyday tasks and impact quality of life in the old and their family members.^6^ Thus, stimulation and preserving this cognitive domain is crucial for this population group.^7^

Executive dysfunction is characterized by the inability to carry out adaptive tasks.^2^ In everyday activities, this is reflected by problems commencing tasks, loss of sense of time, difficulties switching between tasks, controlling impulses, planning and time sequencing, as well as impatience and emotional lability.^8^

Changes in EFs are suggestive of impairment to the frontal lobes or disconnection of the lobes from other brain areas which can lead to deficits in further cognitive functions. Changes in the frontostriatal circuit (neural circuit integrated to the lateral pre-frontal cortex which accesses information related to working memory) are possibly the most significant cause of impaired executive function in elderly with no dementia. Impaired executive function influences memory given its known role in the storage and recall of information.^9^ Although executive problems are not limited to memory tasks, they may represent the main cause of the memory problems experienced by elderly people.^9^

Cognitive intervention may be beneficial for performance of EF tasks. Cognitive training focusing on episodic memory has produced promising results in Brazilian elderly,^10-13^ although no studies involving training of activities associated to EFs are available in Brazil.

In the international literature, the benefits of training focused on working memory (which embraces the EF concept by entailing the storage and processing of information at the same time while requiring mental control). These training programs appear to lead to substantial improvements in this aspect of memory, as well as in executive control.^14,15^ An earlier study assessed the impact of computerized memory training in patients with focal brain injury.^16^

On a national level, no studies assessing the effect of a training program on EF, or reports of training involving working memory, were found. However, a growing number of studies assessing the efficacy of neuropsychological rehabilitation among elderly with significant cognitive impairment are being conducted.^17^

Given the relevance of the theme and current lack of Brazilian studies, the rationale of the present study was to investigate the effect of cognitive training focused on executive functions among healthy elderly with no dementia and/or major depression.

## METHODS

**Participants**. The study involved elderly students of the University of the Third Age (U3A) enrolled on the Challenging Memory workshop at the School of Arts, Sciences and Humanities of the University of São Paulo. The experimental group (EG) comprised 26 elderly submitted to cognitive assessment prior to an intervention consisting of six training sessions following by a post-intervention test assessing the effect of the intervention.

The control group consisted of 17 elderly recruited from other workshops run at the school. The CG completed only the pre and post intervention tests. At study endpoint, control subjects performed one session of the psychoeducational intervention on memory in the aging process and were given the opportunity to take part in the upcoming edition of the same workshop the next semester.

Inclusion criteria were: be older than 55 years of age, have at least two years of schooling, have sufficient sensory (auditory and visual) abilities to take part in the group activities using paper and pencil, have the minimum motor movement needed, show no indication of dementia based on MMSE score, or exhibit suggested major depression on the GDS (>10 points).^18,19^ The following cut-off scores were adopted for schooling level: illiterates, 17 points; 1 to 4 years of schooling, 20 points; 5 to 8 years, 24 points; greater than 8 years, 26 points. These cut-off scores were adapted from Brucki et al.^20^ by subtracting one standard deviation from mean scores found.

**Instruments**. The questionnaire collecting sociodemographic and clinical data included age, income, years of schooling, marital status, general health status, presence of other clinical disease and use of medications. The 15-item Geriatric Depression Scale (GDS),^18,19^ Mini-Mental State Exam (MMSE)^20^ and Story subtest from the Rivermead Behavioural Memory Test (RBMT)^21^ were applied, and versions A and B used to guard against the test-retest effect. Executive functions were assessed by applying the semantic verbal fluency fruit category, and verbal fluency with phonological constraints (FAS), Forward and Backward Digit Span (WAIS-III),^22^ Clock Drawing Test (CDT),^23^ assessed according to criteria proposed by Schulman,^24^ and the Trail Making Test Part A.^25^ The Pfeffer Functional Assessment Questionnaire (PFAQ)^26^ was used assess functional status. Outcome variables included scores on cognitive tests, the GDS and the Pfeffer Functional Assessment Questionnaire (PFAQ).

**Procedures**. At the first session, participants introduced themselves and received time table information. The participants were tested during session two. The pre-test was followed by the six training sessions. At each session, participants performed activities together in a large group with all other members of the EG lasting 40 minutes, during which they were given educational content about memory, cognition and executive functions. In the second half of each session, groups of four to five elderly were formed and activities overseen by a Gerontology graduate for approximately one hour. While in these smaller groups, participants carried our tasks associated to executive functions and attention, as described in Appendix A. After concluding all the training sessions, the elderly were post-tested (ninth session).

Activities were designed to foster the abilities for planning, monitoring, ordering, in addition to inhibiting action and undesired processing. The activities also sought to stimulate abstract reasoning through interpreting proverbs, as well as the attention system by playing cognitive games. Finally, working memory was engaged through problem solving.

**Statistical analyses**. Frequency tables and descriptive statistics such as measurement of position and spread were compiled to provide a profile of the sample. The Chi-square test was employed in order to compare the categorical variables between groups. Kolmogorov-Smirnov's test confirmed that continuous variables did not have a normal distribution (p-value <0.05), and non-parametric tests were therefore applied. Thus, the Mann-Whitney test was used to compare continuous variables between the two groups.

In order to assess evolution of the EG and CG between pre- and post-intervention tests, deltas were calculated (post-test score minus pre-test score). The data were keyed into Epidata version 3., and the SPSS v.17.0 and Stata 11.1 software programs were used for statistical analyses. The level of significance adopted for the statistics tests was 5%, corresponding to a p-value <0.05.

## RESULTS

Results of analyses of sociodemographic data and cognitive variables follows. The participants of the EG and CG proved similar in terms of sociodemographic variables, as well as for MMSE and GDS scores (Table 1).

**Table 1:** Sociodemographic features of study sample.

	EG (26)	CG (17)	p-value
Age	66.20 ± 7.62	67.12 ± 5.67	0.434^a^
Schooling	10.08 ± 5.66	8.88 ± 3.53	0.493^a^
Income - minimum wages	3.89 ± 1.68	3.82 ± 1.78	0.970^a^
MMSE	27.15 ± 2.33	27.65 ± 2.40	0.413^a^
GDS	2.19 ± 1.79	2.06 ± 2.04	0.685^a^
No. of women	22	12	0.269^b^

a p-value refers to Mann-Whitney test for independent samples;

b p-value refers to the chi square test. MMSE: Mini-Mental State Exam; GDS: Geriatric Depression Scale.

Table 2 shows significant improvement in the CG on the Story and Digit Span at post-test. However, worse performance was seen in the CG on verbal fluency tasks at post testing. Scores in the EG remained the same at pre and post tests. Table 3 depicts the results of the analysis of delta values (post-test score minus pre-test score for cognitive variables) showing similar results at baseline and endpoint.

**Table 2:** Comparison of pre- and post-intervention test results of EG and CG for outcome variables.

Variables	Time	Groups		p value^a^
		**EG (26)**	**CG (17)**	
**GDS**	Pre-Test	2.19 ± 1.79	2.06 ± 2.05	0.685
	Post-Test	1.92 ± 1.44	3.00 ± 2.12	0.091
**MMSE**	Pre-Test	27.15 ± 2.33	27.65 ± 2.40	0.413
	Post-Test	27.08 ± 2.26	27.57 ± 1.74	0.547
**Story RBMTImmediate Recall**	Pre-Test	5.00 ± 2.49	4.68 ± 2.26	0.662
	Post-Test	5.48 ± 2.48	7.85 ± 3.39	0.021
**Verbal Fluency**				
**Fruits**	Pre-Test	12.92 ± 2.84	12.82 ± 3.40	0.548
	Post-Test	13.50 ± 3.30	11.94 ± 3.17	0.072
**FAS - Total 3 letters**	Pre-Test	45.46 ± 13.64	47.24 ± 14.89	0.881
	Post-Test	47.19 ± 13.16	38.12 ± 12.70	0.016
**Story RBMTDelayed Recall**	Pre-Test	4.54 ± 2.73	4.06 ± 2.17	0.699
	Post-Test	5.35 ± 2.54	7.32 ± 3.41	0.052
**Clock Drawing**	Pre-Test	4.00 ± 1.15	4.06 ± 1.14	0.827
	Post-Test	3.96 ± 1.23	4.00 ± 0.79	0.640
**Total Digit Span**	Pre-Test	12.27 ± 3.29	12.24 ± 2.91	0.764
	Post-Test	13.15 ± 4.08	18.53 ± 6.15	0.004
**Trail-making A**	Pre-Test	62.69 ± 34.23	57.29 ± 32.20	0.881
	Post-Test	52.54 ± 26.10	43.65 ± 23.74	0.176
**PFAQ**	Pre-Test	1.15 ± 1.52	0.35 ± 0.61	0.077
	Post-Test	0.62 ± 1.13	0.65 ± 0.93	0.665

a Mann-Whitney test for independent samples. MMSE: Mini-Mental State Exam; GDS: Geriatric Depression Scale; RBMT: Rivermead Behavioural Memory Test; FAS: Phonemic Verbal Fluency; PFAQ: Pfeffer Functional Assessment Questionnaire..

**Table 3:** Deltas (post-test scores minus pre-test scores) for performance of participants.

Variables	Groups	Time		Delta
		**Pre-Test**	**Post-Test**	
**GDS**	EG (26)	2.19 ± 1.79	1.92 ± 1.44	-0.27 ± 1.95
	CG (17)	2.06 ± 2.05	3.00 ± 2.12	0.94 ± 1.89
**MMSE**	EG (26)	27.15 ± 2.33	27.08 ± 2.26	-0.08 ± 3.47
	CG (17)	27.65 ± 2.40	27.57 ± 1.74	0.29 ± 2.40
**StoryImmediate Recall**	EG (26)	5.00 ± 2.49	5.48 ± 2.48	0.48 ± 2.92^a^
	CG (17)	4.68 ± 2.26	7.85 ± 3.39	3.18 ± 3.71^a^
**Verbal Fluency**				
**Fruits**	EG (26)	12.92 ± 2.84	13.50 ± 3.30	0.58 ± 3.25
	CG (17)	12.82 ± 3.40	11.94 ± 3.17	-0.88 ± 4.09
**FAS - Total 3 letters**	EG (26)	45.46 ± 13.64	47.19 ± 13.16	1.73 ± 9.04^a^
	CG (17)	47.24 ± 14.89	38.12 ± 12.70	-9.12 ± 18.70^a^
**StoryDelayed Recall**	EG (26)	4.54 ± 2.73	5.35 ± 2.54	0.81 ± 3.17
	CG (17)	4.06 ± 2.17	7.32 ± 3.41	3.26 ± 3.99
**Clock Drawing**	EG (26)	4.00 ± 1.15	3.96 ± 1.23	-0.00 ± 0.95
	CG (17)	4.06 ± 1.14	4.00 ± 0.79	-0.06 ± 1.09
**Total Digit Span**	EG (26)	12.27 ± 3.29	13.15 ± 4.08	0.88 ± 5.14^a^
	CG (17)	12.24 ± 2.91	18.53 ± 6.15	6.29 ± 5.68^a^
**Trail-making A**	EG (26)	62.69 ± 34.23	52.54 ± 26.10	-10.15 ± 22.28
	CG (17)	57.29 ± 32.20	43.65 ± 23.74	-13.65 ± 27.29
**PFAQ**	EG (26)	1.15 ± 1.52	0.62 ± 1.13	-0.54 ± 1.42
	CG (17)	0.35 ± 0.61	0.65 ± 0.93	0.29 ± 1.10

a Mann-Whitney test for independent samples. MMSE: Mini-Mental State Exam; GDS: Geriatric Depression Scale; RBMT: Rivermead Behavioural Memory Test; FAS: Phonemic Verbal Fluency; PFAQ: Pfeffer Functional Assessment Questionnaire.

## DISCUSSION

The aim of the present study was to test the efficacy of cognitive training focused on executive functions given to healthy elderly with no dementia and/or major depression who frequented an Open University for the Third Age. The study sought to run a cognitive intervention protocol design to stimulate skills associated to the EF concept.

The results indicated greater improvement in the CG (Story and Digit span) than the EG at the post-intervention test. It should be noted that the EG has only a small number of sessions which may not have proven sufficient to exert a significant effect on performance of EF tasks. One could conclude that the changes seen in the CG were attributed to the test-retest effect but the same effect was not observed in the EG, and different versions of the cognitive test, where available, were used in a bid to minimize this effect. An alternative explanation for the improvement seen in the CG may be the influence of subjects' participation in other workshops held at the University. Elderly from both EG and CG may have taken part in other activities during the semester of the study, and these may have led to a difference in the pattern of intellectual, physical and social stimulation. Future studies with protocols that include questions assessing the profile of participation in other activities inside and outside University are needed to confirm this hypothesis.

With regard to the absence of impact seen on the assessment of functioning by the PFAQ, it is noteworthy that the groups studied comprised independent, socially engaged elderly without functional changes. Thus, the results suggest a ceiling effect for this variable. Nevertheless, earlier studies have reported that pursuing educational activities, such as those offered at universities for the third age and community centers, can contribute toward maintaining independence of elders.^33-36^

A number of previous studies have confirmed a positive effect of working memory training in older adults.^15,27-30^ Some studies have explored the impact of cognitive training of executive functions, allied with ecological activities, among individuals with chemical dependence, schizophrenia and children with Attention Deficit Hyperactivity Disorder.^31,32^ The positive results of these studies reported in the literature suggest that the performance improvement on EF observed in clinical samples may be more common when performance on neuropsychological tests at baseline show greater deviation from normal levels compared to healthy individuals. Thus, the absence of changes after training on EF may be viewed as a finding inherent to stimulation studies involving healthy participants, as proposed by Buschkuehl et al.^29^

Studies on cognitive plasticity have proved a fertile field for basic research, since they yield essential information about human aging. At the same time, these studies have important implications for clinical practice of professionals dealing with the elderly population since the optimizing of cognition is linked to health, autonomy and independence. Ramos^38^ conducted an epidemiological study and found functional dependency and cognitive decline to be associated to mortality, while highlighting that these factors can respond to interventions.

The present study constituted a pilot study aimed at devising a protocol for training executive functions among older adults. The intervention proposed can be refined in future studies given that some of the activities included in the current protocol involve memory subsystems that are not exclusively EFs. Other important limitations of this study include the relatively small sample and the low number of sessions run. Finally, the study has a methodological limitation in that participants were not randomly distributed. Future studies could be based on a longer intervention with longitudinal follow up to detect potential maintenance of effects.

## APPENDIX A - DESCRIPTION OF TRAINING ACTIVITIES ON EXECUTIVE FUNCTIONS

**Table:**

Session 1	In large group (60 minutes):	Introductory class on cognition and aging. Discussion on changes in memory as part of the normal aging process. Reports from participants on day-to-day successes related to organization, planning, temporal-spatial orientation, problem solving and memory.
	In small groups (60 minutes):	A short text about the environment was read and participants counted mentally how many time a given word appeared in the text. Verbal fluency activity with semantic and phonological constraints with animals beginning with the letter A, C and L. Reading of sequences of three words with repetition in reverse order, reading of word sequences for placing in alphabetic order by participants. Verbal fluency activity and memorization, with each participant stating what they would take on a trip after repeating what their peers would take along.
**Session 2**	In large group (60 minutes):	Introductory class on attention and its importance for memorizing and recalling new information.
	In small groups (60 minutes):	Auditory attention exercise, in which participants listened to a list of six news headlines about healthy life habits and had to decide whether each was a good or bad news item by raising their right or left hand, respectively. Afterwards, participants were expected to recall the last word of each headline. Visual attention exercise, in which participants spotted squares in a sheet full of small geometric figures.
		A game of noughts and crosses was played in groups to train executive functions based on working memory in which participants were split into two group, one representing crosses (X) and the other noughts (O). Players uncovered the questions answering them correctly to win the game. At each round, one participant represented the group to give the answer to the question and together with fellow players picked a square to place the symbol strategically to try and win the game.
		Performing a task placing words in chronological order, such as seasons of the year or phases of the life cycle. Placing sequences of a story in order, where participants organized the phrase in an effort to construct stories. Phonological domain when participants uttered a word for instance, "boneca" (doll), and the next player had to come up with another word beginning with the last syllable of the word given by the previous player e.g. "caneca" (mug). Verbal fluency activity performed in groups with semantic constraints.
**Session 3**	In large group (60 minutes):	Brief introduction to the concept of executive functions and neural correlates. Reports from participants on day-to-day successes related to organization, planning, temporal-spatial orientation, problem solving and memory.
	In small groups (60 minutes):	STOP activity, dealing with verbal fluency with categorical and phonological constraints. Task of reverse order in which participants read word sequences and repeated them backwards. Auditory spot-the-difference task in which players heard a group of words and had to identify if they were the same or different.
**Session 4**	In large group:	Class on the concept of executive functions and the aging process.
	In small groups:	Visual attention activity, performed with a spot the seven differences game played in pairs. Chronological ordering of stories. Verbal fluency STOP activity with six rounds, dealing with verbal fluency and categorical and phonological constraints. Slowpace dance activities with simulations and repetition of simple steps, showing how many abilities are deployed in learning the steps (involvement of attention, planning, organisation and working memory).
**Session 5**	In large group:	Neuroanatomical aspects related to executive functions (continuation of previous class) and examples of pathologies related to executive dysfunction.
	In small group:	Attention activity, spot the seven differences game. Auditory attention task to identify whether words spoken aloud are the same or different. Instruction for the last task: you are going to hear a group of three words. If the three words are the same, write A. If they are different then write B. If two are the same and one is different, write C. Examples: Soap, detergent, orange. Answer: C; Jabuticaba fruit, honeydew melon, banana; Answer: A. Dog, cloud, book; Answer: B, and so forth alternating according to the items and categories chosen. Another task performed was the printed activity, Maze Game. Here a soldier (photo of a man in uniform) needed to find a way out from a maze to avoid an ambush with several wrong paths and only one correct one.
**Session 6**	In large group:	Review of theoretical content presented
	In small groups:	Task simulating change in which participants selected product packs labelled with a price tag and had to mentally tally the total purchase, pay and check whether the change given was correct or incorrect. STOP activity, involving verbal fluency with categorical and phonological constraints. To conclude, participants were shown three boards depicting figures and situations in which the elderly had to find a specific object. The idea was to exploit the attention system and reasoning of the participants. Miming activity, elderly volunteers, picked a situation to simulate and peers had to uncover the challenge to try and find out what the challenge was about.
